# Panspinal Epidural Abscess: A Devastating Complication of Group B Streptococcal Bacteremia

**DOI:** 10.1155/2022/5028335

**Published:** 2022-05-20

**Authors:** Rehmat Ullah Awan, Ambreen Nabeel, Mohammed Alsaggaf

**Affiliations:** ^1^Ochsner Rush, Meridian, Mississippi, USA; ^2^Ochsner Health System, New Orleans, Louisiana, USA

## Abstract

**Background:**

SEAs are infrequent; however, panspinal infections are even rarer, especially when GBS infection is involved. The cornerstone of treatment is based on early diagnosis and use of targeted antimicrobial therapy; in case of cord compression or neurological compromise, urgent surgical intervention should be pursued. Overall, it is an infrequent condition and therefore requires prospective multicenter studies. *Case Presentation*. We describe a case who presented with diabetic lower extremity wounds; however, soon the patient developed bowel and bladder incontinence in the setting of back pain, secondary to panspinal epidural abscess. The patient's case is unique in two aspects: firstly, it is panspinal, and secondly, its causative agent is GBS.

**Conclusion:**

Prompt diagnosis of SEA is critical in the preservation of neurological function. Anyone presenting with fevers, back pain, and neurological changes should have urgent MRI evaluation of the spine.

## 1. Background

Spinal epidural abscesses (SEAs) are rare; however, delay in the diagnosis and treatment can be devastating with irreversible neurological deficits, and mortality has been reported up to 32% [1]. People with weakened immune system or having chronic conditions such as diabetes, renal failure, and alcoholism are at risk of developing SEA [2]. SEAs are usually localized to a specific spinal region; however, very rarely they can affect all the spinal levels causing panspinal epidural abscess [3]. The GBS incidence in nonpregnant adults is increasing, especially in chronically ill patients [4]. However, GBS causing SEA is reported in only a handful of cases. Due to the rarity of panspinal epidural abscess, there are no clear treatment guidelines, and therefore, more studies are needed to understand this disease in depth.

## 2. Case Presentation

A 45-year-old native American presented to our emergency department with right lower extremity pain due to necrotic right foot wounds. He has a history of poorly controlled diabetes, alcoholism, right transmetatarsal amputation, and right thumb amputation. Recently, the patient had developed increasing right foot pain for which he visited with his elders to a witch doctor who performed linear lacerations on his leg to relieve pain. Upon presentation, he was hemodynamically stable, no positive finding on exam other than purulent and foul-smelling drainage from his right foot wounds, the linear cuts were clean with no signs of infection, and all reviews of systems and exam were normal other than what was stated above. An X-ray image showed gas gangrene in the right foot near his transmetatarsal amputation site. His labs revealed Na 129 mmol/L, Cr 2.58 mg/dL, BUN 46 mg/dL, lactic acid 5.1 mmol/L, WBC 22.01K/uL, Hb 9.4 g/dL, Plt 482K/uL, HbA1C 12.9%, ALK Phos 313 U/L, ALT 3 U/L, AST 23 U/L, albumin 0.8 g/dL, total protein 5.8 g/dL, INR 1.46, HIV and Hep C serologies negative, CRP 147.9 mg/L, and procalcitonin 1.72 ng/mL. Blood cultures and wound cultures on admission later turned out to be positive for *Streptococcus agalactiae* (Group B).

**Figure 1:**
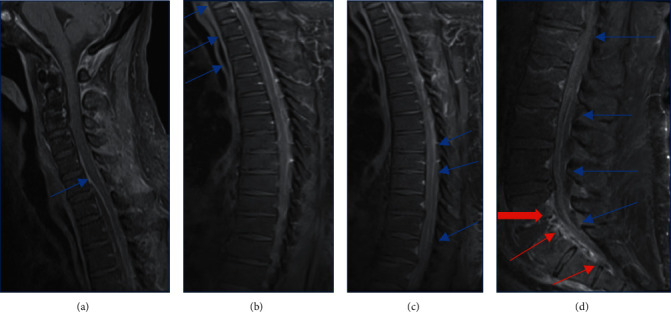
T1-weighted MRI scans: (a) anterior epidural rim enhancing collection extending from C6-T4 (below the image field). The starting point of the abscess is marked by a blue arrow. There is posterior displacement of the spinal cord with significant narrowing of the spinal canal. (b) Extension of the anterior epidural abscess from the cervical spine to the T4 level (blues arrows). There is a mass effect and flattening of an anterior aspect of the spinal cord. (c) Posterior epidural minimally enhancing collection extending from T7-T8 level to L5-S1. (d) The poster epidural collection extending from the thoracic spine to L5-S1 (blue arrows). L5-S1 osteomyelitis-discitis (thick red arrow) and anterior epidural abscess extending from the same level to the lower sacrum.

The patient was started on vancomycin and piperacillin tazobactam empirically and was then taken to the OR urgently for guillotine amputation of the right foot for source control. The patient was taken back to the OR after 4 days for right sided below the knee amputation for a definitive closure. His repeat blood cultures were negative. Transthoracic echocardiogram was negative for any endocarditis. He started to work with physical and occupational therapy; however, he began to complain of intermittent lower back pain. Despite appropriate source control (right BKA), broad-spectrum intravenous antibiotics (vancomycin and zosyn), and negative blood cultures, the patient's WBC count remained very high >20K/uL. On day two of his second surgery, the patient acutely developed bowel and bladder incontinence, and upon evaluation, his motor and sensory functions were preserved in lower and upper extremities, though he did have saddle anesthesia. Urgent MRI spine was performed which showed multifocal epidural collections throughout the cervical, thoracic, and lumbar spine with various degrees of cord deformity and displacement within the spinal canal (Figure 1). Neurosurgery evaluated the patient, and he was taken to the OR for C7-T2 left hemilaminectomies, T9-T10 left hemilaminectomies, and L3 laminectomy for evacuation of epidural abscess. The cultures taken in the OR returned positive for *Streptococcus agalactiae* (Group B). The patient was started on intravenous ceftriaxone 2 g twice a day on infectious disease's recommendation for a total of 8 weeks. The patient's WBC count started to decrease rapidly and returned to normal on post-op day 3. The patient did not appear to have any significant motor or sensory deficits, though continued to have neurogenic bladder. He was transferred to the lower level of care for wound care, physical therapy, and continued intravenous antibiotics.

## 3. Discussion

SEAs are rare, with an incidence at 0.2-3/10,000 hospital admissions [2], and if left untreated, they can lead to increased morbidity and mortality. The most common causative agent is *Staphylococcus aureus* [5],and the most common site is the lumbar spine [6]. SEAs usually present as back pain, fevers, and neurological compromise, and MRI spine is sufficient for diagnosis [3]. We report an unusual case of multilevel/panspinal epidural abscess involving the cervical, thoracic, and lumbar spine secondary to GBS; this is the second case being reported with panspinal epidural abscess caused by GBS and the first one being reported by David Wen et al. [1]. Our case is unique in terms of management due to combination of medical and surgical intervention.

GBS is a Gram-positive coccus that commonly colonizes the urogenital system, and it typically causes UTIs, skin infections, and bacteremia; however, it is not common. The incidence of systemic GBS infection is increasing in nonpregnant adult patients with mortality rising to 21% [4], and metastatic infectious foci tend to further complicate clinical outcomes [7]. Adults with GBS infections should all be screened for immunocompromising conditions as their presence in healthy adults is uncommon [2, 8, 9], as seen in our case who had poor glycemic control along with alcohol abuse; both are risk factors for a poor immune system.

Discitis and SEAs secondary to GBS are reported only a handful of times in the literature; however, panspinal involvement is even rarer, and therefore, no clear guidelines are set for their management. The literature is not quite clear about the management of SEAs overall, with some experts favoring combination therapy (antibiotics and surgery) whilst some recommending conservative therapy [10, 11]. A case series review concluded that panspinal infection is better treated surgically in conjunction with antimicrobial therapy rather than antibiotics alone [3]. Regardless of the mode of treatment, it is important to sample the abscess site either operatively or through CT-guided imaging to establish targeted treatment plan. GBS strains are mostly pan-sensitive; however, emerging resistance to erythromycin and clindamycin has been reported. Therefore, treatment of choice remains penicillin-derived antibiotics [9, 12]. Duration of antibiotics is anywhere between 8 and 12 weeks [1, 3, 13].

In terms of initial evaluation, thorough neurological exam, WBC count, and C-reactive protein (CRP) are very important, especially an elevated WBC count and CRP are associated with poor outcomes [14]. In our case, the patient's WBC count did not improve until surgical abscess evacuation was carried out, and similarly, his CRP on admission was extremely high at 147.9 mg/L which decreased to 18 mg/L 9 days after surgery.

## 4. Conclusion

Prompt diagnosis of SEA is critical in the preservation of neurological function. Anyone presenting with fevers, back pain, and neurological changes should have urgent MRI evaluation of the spine. Moreover, CRP and the WBC count should be trended to guide the effectiveness of therapy. The GBS infection rate is rising, and early blood and tissue cultures can help clinicians formulate a targeted antimicrobial therapy, which can prevent further spread of the infection and improve outcomes.

## Data Availability

For data search, see references in the manuscript, and the patient's information is only in our health-care systems EMR and is protected.

## Abbreviations

SEA: Spinal epidural abscess
WBC: White blood count
CRP: C-reactive protein
GBS: Group B streptococcus
BKA: Below knee amputation
MRI: Magnetic resonance imaging
ALT: Alanine transferase
AST: Aspartate transferase
ALP: Alkaline phosphatase
UTI: Urinary tract infection.
